# TbGT8 is a bifunctional glycosyltransferase that elaborates *N*-linked glycans on a protein phosphatase AcP115 and a GPI-anchor modifying glycan in *Trypanosoma brucei*

**DOI:** 10.1016/j.parint.2014.01.007

**Published:** 2014-06

**Authors:** Masayuki Nakanishi, Moe Karasudani, Takahiro Shiraishi, Kazunori Hashida, Mami Hino, Michael A.J. Ferguson, Hiroshi Nomoto

**Affiliations:** a Laboratory of Biochemistry, College of Pharmaceutical Sciences, Matsuyama University, Matsuyama, Ehime 790-8578, Japan; b Division of Biological Chemistry and Drug Discovery, College of Life Sciences, University of Dundee, Dundee DD1 5EH, United Kingdom

**Keywords:** CBB, Coomassie brilliant blue, cKO, conditional double knockout, FP, flagellar pocket and lysosome/endosome system, GlcNAc, *N*-acetylglucosamine, GPI, glycosylphosphatidylinositol, HA, hemagglutinin epitope, LacNAc, *N*-acetyllactosamine, PBS, phosphate buffered saline, PNGase, peptide *N*-glycosidase, VSG, variant surface glycoprotein, Glycosyltransferase, *Trypanosoma brucei*, *N*-linked glycan, GPI-anchor, Tomato lectin

## Abstract

The procyclic form of *Trypanosoma brucei* expresses procyclin surface glycoproteins with unusual glycosylphosphatidylinositol-anchor side chain structures that contain branched *N*-acetyllactosamine and lacto-*N-*biose units. The glycosyltransferase TbGT8 is involved in the synthesis of the branched side chain through its UDP-GlcNAc: βGal β1-3*N*-acetylglucosaminyltransferase activity. Here, we explored the role of TbGT8 in the mammalian bloodstream form of the parasite with a tetracycline-inducible conditional null *T. brucei* mutant for *TbGT8*. Under non-permissive conditions, the mutant showed significantly reduced binding to tomato lectin, which recognizes poly-*N*-acetyllactosamine-containing glycans. Lectin pull-down assays revealed differences between the wild type and *TbGT8* null-mutant *T. brucei*, notably the absence of a broad protein band with an approximate molecular weight of 110 kDa in the mutant lysate. Proteomic analysis revealed that the band contained several glycoproteins, including the acidic ecto-protein phosphatase AcP115, a stage-specific glycoprotein in the bloodstream form of *T. brucei*. Western blotting with an anti-AcP115 antibody revealed that AcP115 was approximately 10 kDa smaller in the mutant. Enzymatic de-*N*-glycosylation demonstrated that the underlying protein cores were the same, suggesting that the 10-kDa difference was due to differences in *N*-linked glycans. Immunofluorescence microscopy revealed the colocalization of hemagglutinin epitope-tagged TbGT8 and the Golgi-associated protein GRASP. These data suggest that TbGT8 is involved in the construction of complex poly-*N*-acetyllactosamine-containing type *N*-linked and GPI-linked glycans in the Golgi of the bloodstream and procyclic parasite forms, respectively.

## Highlights

• TbGT8 is involved in *N*-linked glycan synthesis in the bloodstream form.
• AcP115 is a target glycoprotein of TbGT8-dependent glycan processing.
• TbGT8 is localized in the Golgi and modified by *N*-linked glycan(s).

## Graphical abstract

**Figure:**
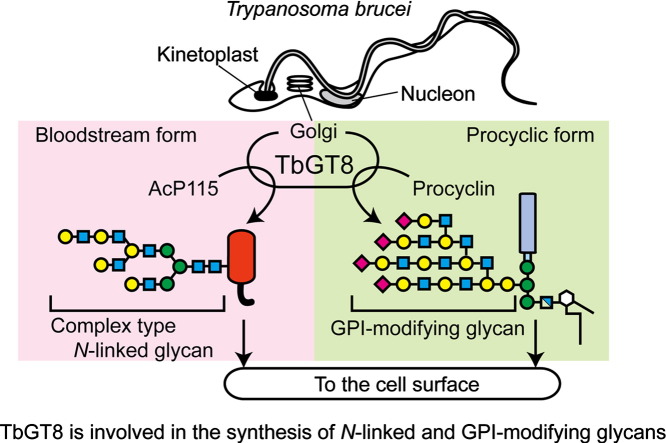

## 1 Introduction

Glycans that are covalently attached to proteins can modulate protein properties such as folding, activity, stability, trafficking, and recognition [1]. These functions are known to be important to multicellular organisms, which require highly organized intercellular interactions, and therefore the expression of incomplete or modified glycans often leads to embryonic death or congenital disease [2,3]. However, our knowledge about glycan functions in unicellular parasites such as *Trypanosoma brucei* is less advanced, despite the fact that these organisms synthesize an impressive range of glycoconjugates [4].

*T. brucei* causes African sleeping sickness in humans and nagana in cattle. The parasite has a complex life cycle between its mammalian hosts and tsetse fly vectors. The parasite glycans have been extensively analyzed in both the mammalian bloodstream and insect-dwelling procyclic forms of the parasite, and these studies have revealed the existence of asparagine (*N*)-linked oligomannose, paucimannose, and complex type glycans, as well as protein GPI-anchors bearing complex glycan side chains [5–12].

The bloodstream form of the parasite is covered with a dense layer of a single-species variant surface glycoprotein (VSG) that protects it against the host's innate and acquired immune systems [6]. The protection is achieved by forming a physical barrier against the approach of complement molecules to the plasma membrane and by undergoing antigenic variation. Molecular modeling predicts that the *N*-linked glycans on VSG occupy inter-VSG spaces to facilitate the shielding of invariant surface antigens [13]. Other glycoproteins such as invariant surface glycoproteins are also found on the plasma membrane, although their functions are unclear. In addition, several other glycoproteins are known to be located in the flagellar pocket (FP), a small invagination of the cell surface where the flagellum exits the cytoplasm. FP is the only known site for endocytosis and secretion within the lysosomal/endosomal recycling system. The transferrin receptor, for example, is a FP-residing heterodimeric glycoprotein that contains a single GPI-anchor and 8*N*-glycosylation sequons. Their *N*-linked glycans are thought to secure sufficient space for the binding of transferrin to its receptor [14]. The lysosome-associated membrane protein p67, also partly located in the FP, is heavily *N*-glycosylated and is thought to contain poly-*N*-acetyllactosamine (LacNAc) structures [15]. Such glycans act as internalization signals for the parasite's endocytosis system through a putative lectin-like receptor in the FP [16], although this model has been questioned recently [14].

The procyclic parasite form proliferates in the midgut of the tsetse fly and is coated with GPI-anchored procyclin glycoproteins [6], free GPI glycolipids [17–19], and a partially characterized high-molecular-weight glycoconjugate [20,21]. The procyclin GPI-anchor contains a large branched side-chain that comprises LacNAc and lacto-*N*-biose units terminated with α2-3-linked sialic acid residues [8], which appear to play a critical role in the successful colonization of the tsetse fly [18]. A shorter GPI-anchor side chain is produced when *TbGT8* is deleted from the parasites. A detailed structural analysis of the GPI-anchors revealed that TbGT8 is involved in the side chain synthesis through its UDP-GlcNAc: βGal β1-3*N*-acetylglucosaminyltransferase activity [22].

Deletion of *TbGT8* in the bloodstream form results in reduced reactivity to the tomato lectin [22]. However, the entities of the glycosylated molecules have not yet been elucidated. In this paper, we demonstrate that in the bloodstream form, TbGT8 is involved in the synthesis of *N*-linked glycans that attach to an acidic phosphatase and colocalizes with a Golgi marker protein.

## 2 Materials and methods

### 2.1 Cultivation of trypanosomes

A bloodstream form of *T. brucei* (strain 427, variant 221) that had been genetically modified to express T7 RNA polymerase and the tetracycline repressor was cultured in HMI-9 medium containing 2.5 μg/mL of G418 at 37 °C in a 5% CO_2_ incubator. This strain is referred to as the “wild type” in this paper.

### 2.2 Establishment of the conditional knockout (cKO) strain

A strain that expresses hemagglutinin epitope (HA)-tagged TbGT8 (TbGT8HA) upon the addition of tetracycline was established as follows. The *TbGT8* coding sequence (Tb927.10.12290) was amplified by PCR using the primers HindIII-GT8f (5′-ACaagcttCACCATGGTTGGACAAATTTTGAG-3′) and BamHI-GT8r (5′-CTggatccCACCGCTTGCCGCATGTTGCG-3′), and was cloned into a variant of the pLew100 expression vector [23] at the *Hin*dIII and *Bam*HI sites. The variant vector contained the HA-tag DNA coding sequence at the *Bam*HI site for C-terminal fusion of the HA-tag. The construct was linearized by *Not*I digestion and electroporated into the *TbGT8* null mutant (∆TbGT8::puromycin acetyl transferase gene/∆TbGT8::hygromycin phosphotransferase gene), which was established from the wild type strain as previously described [22]. The parasites were selected in the presence of 2.5 μg/mL of phleomycin. The established strain is designated “cKO” in this paper. Permissive and non-permissive conditions to induce *TbGT8HA* expression indicate cultivation conditions with and without 1 μg/mL of tetracycline, respectively.

### 2.3 Enrichment of the tomato lectin-binding protein

The cultured parasites (1 × 10^8^ cells) were washed thrice with phosphate buffered saline (PBS) and lysed in 1.5 mL of RIPA (−) buffer [50 mM Tris HCl (pH 8.0) containing 0.15 M NaCl, 1% Nonidet P40, 0.5% sodium deoxycholate, and protease inhibitor cocktail] for 10 min on ice. The supernatants were collected by centrifugation at 18,900 ×*g* for 10 min at 4 °C. Subsequently, 100 μL of tomato lectin–agarose slurry (Vector Laboratories, Burlingame, CA) in RIPA (−) was added to the supernatant, and the mixture was incubated for 2 h at 4 °C on a rotating platform, followed by 3 washes with 0.4 mL of RIPA (−). The bound molecules were eluted with 0.3 mL of RIPA (−) containing chitin hydrolysate (Vector Laboratories). The eluent was subsequently mixed with 0.9 mL of acetone containing 10% trichloroacetic acid and 0.07% 2-mercaptoethanol, followed by incubation for 1 h at − 30 °C for protein precipitation. The precipitate was collected by centrifugation at 18,900 ×*g* for 5 min at 4 °C, followed by a wash with 0.3 mL of acetone that contained 0.07% 2-mercaptoethanol. The protein samples obtained were separated on a 10% NuPAGE gel (Life Technologies, Carlsbad, CA) and stained with the Colloidal Blue Staining kit (Life Technologies). Protein bands were cut from the gel for LC–MS/MS protein identification at the Proteomics and Mass Spectrometry Facility, College of Life Sciences, University of Dundee.

### 2.4 Raising polyclonal antibodies against AcP115, TbGRASP, and TbBiP

The DNA sequence that encoded Ser24–Ile347 of AcP115 (Tb927.5.630) was amplified from the wild type strain genomic DNA by PCR using the primers HindIII-APf (5′-ACaagcttTCGAGCAGCGATGCGCAAC-3′) and BamHI-APr (5′-CGTggatccGATATCGTCAACGGAAAT-3′). After abolishing the internal *Hin*dIII site, the DNA was cloned into a variant of pQE30 (QIAGEN, Hilden, Germany) plasmid at the *Hin*dIII and *Bam*HI sites, where the 2 sites were exchanged with each other. The full-length TbGRASP coding sequence (Tb927.11.2660) was obtained by PCR using the primers BamHI-GRf (5′-CAAggatccATGGGACAGGGGAAAAGCG-3′) and HindIII-GRr (5′-GTCaagcttAGCGAGATGGTGTGGCTG-3′). The DNA was cloned into the pQE30 plasmid at the *Bam*HI and *Hin*dIII sites. These constructs were used to produce N-terminal His-tagged recombinant proteins in *Escherichia coli*, according to the manufacturer's instructions. In both cases, the bacteria were homogenized by sonication in a buffer that contained 20 mM Tris HCl (pH 7.5) and 0.3 M NaCl. The homogenates were centrifuged for 15 min at 15,000 ×*g*, and the resulting supernatants were first applied to a Co^2 +^ Talon® column and subsequently to Sephacryl S200HR gel filtration column that was pre-equilibrated with the same buffer used in the His-tagged protein purification. Polyclonal antibodies were raised against the purified proteins in rabbits according to a 77-day protocol (Operon Biotechnologies, Huntsville, AL). A rabbit polyclonal antibody against TbBiP was raised against a keyhole limpet hemocyanin-conjugated peptide that corresponded to amino acids 635–653 of TbBiP (Tb927.11.7460), according to a 77-day protocol (Sigma-Aldrich, St. Louis, MO).

### 2.5 SDS–PAGE, lectin blotting, and western blotting

The parasite lysates were treated with or without peptide *N*-glycosidase (PNGase) F (Roche Diagnostics, Basel, Switzerland), separated on SDS–PAGE gels, and transferred onto BioTrace PVDF membranes (PALL, Port Washington, NY). The membranes were blocked for 1 h with the protein-free PVDF Blocking Reagent (TOYOBO, Osaka, Japan). For lectin blotting, the membranes were incubated with 2 μg/mL of biotin-conjugated tomato lectin (Vector Laboratories) in 10 mM Tris HCl (pH 7.5) with 150 mM NaCl and 0.05% Tween20 (TBST); washed thrice with TBST; incubated for 30 min with Vectastain ABC reagent (Vector Laboratories); washed thrice with TBST; and developed with an enhanced chemiluminescent reagent (Promega, Fitchburg, WI), according to the manufacturer's instructions. For Western blotting, the antigen was detected with a 1:2000 dilution of polyclonal antisera in CanGetSignal reagent I (TOYOBO), followed by an HRP-conjugated anti-rabbit IgG (Promega) diluted in 1:4000 in CanGetSignal reagent II (TOYOBO).

### 2.6 Fluorescence microscopy

The bloodstream forms of *T. brucei* were washed with PBS and resuspended and fixed in PBS containing 4% paraformaldehyde for 5 min at room temperature. The fixed parasites were washed with PBS and allowed to attach to coverslips for 5 min. The coverslips were submerged in PBS containing 0.1% Nonidet P40 for 5 min to permeabilize the cells. Subsequently, PBS containing 3% bovine serum albumin was added for a 1-h blocking period. After blocking, the cover slips were incubated for 1 h with a 1:1000 dilution of rabbit polyclonal anti-TbGRASP and 1 μg/mL of rat monoclonal anti-HA 3 F10 (Roche Diagnostics) in CanGetSignal Immunostain A (TOYOBO), followed by three 10-min washes in PBS containing 0.5% BSA. Following this, the cover slips were washed thrice in PBS containing 0.5% BSA and incubated for 1 h with a 1:1000 dilution of AlexaFluor 594-conjugated anti-rabbit IgG (Life Technologies) and 2 μg/mL of AlexaFluor 488-conjugated anti-rat IgG (Cell Signaling Technology, Danvers, MA) in CanGetSignal Immunostain B. The coverslips were subsequently washed thrice and mounted in antifade mounting solution containing DAPI (Vector Laboratories). Images were obtained with a confocal laser scanning microscope 510 (Zeiss). All images were obtained and processed under the similar settings.

## 3 Results

### 3.1 Conditional expression of TbGT8HA

**Fig. 1:**
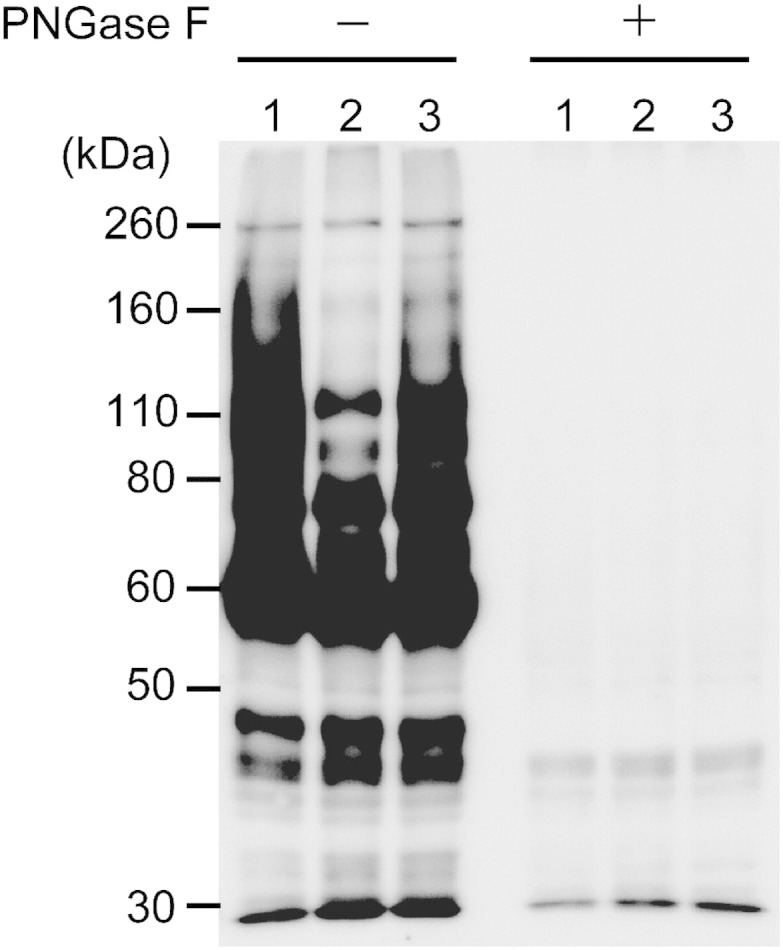
TbGT8 is involved in *N-*linked glycan synthesis in the bloodstream form of *Trypanosoma brucei*. The whole parasite lysates in RIPA (−) were subjected to SDS–PAGE, transferred to PVDF membranes, and detected with tomato lectin. Lanes 1–3 are the lysates of wild type, cKO under non-permissive, and cKO under permissive conditions, respectively. The loss of TbGT8 expression selectively reduced the smear signal at approximately 110 kDa. PNGase F treatment eliminated the lectin binding.

The cKO strain was established to express HA-tagged TbGT8 (TbGT8HA) in response to the addition of tetracycline. The strain's genetic background lacks both of the original *TbGT8* alleles, but the genome contains an inducible gene cassette for TbGT8HA expression. The conditional expression and functionality of TbGT8HA were investigated with tomato lectin blotting. The wild type lysate showed a dense smear pattern at approximately 110 kDa on the lectin blot, while the cKO lysate showed a significant loss of this pattern under non-permissive conditions, i.e., in the absence of tetracycline (Fig. 1). In contrast, permissive conditions modestly restored the smear pattern in the cKO lysates. Treatment of the lysates with PNGase F, which removes *N*-linked glycans from proteins, resulted in a near-complete loss of lectin binding. These results demonstrate that TbGT8 is involved in the synthesis of *N*-linked glycans of approximately 110 kDa glycoproteins and is functional even if an HA-tag is fused to its C-terminus. The controlled protein expression of TbGT8HA under permissive conditions will be proven later (Section 3.4) by Western blotting.

### 3.2 Proteomic identification of tomato lectin-binding proteins

**Fig. 2:**
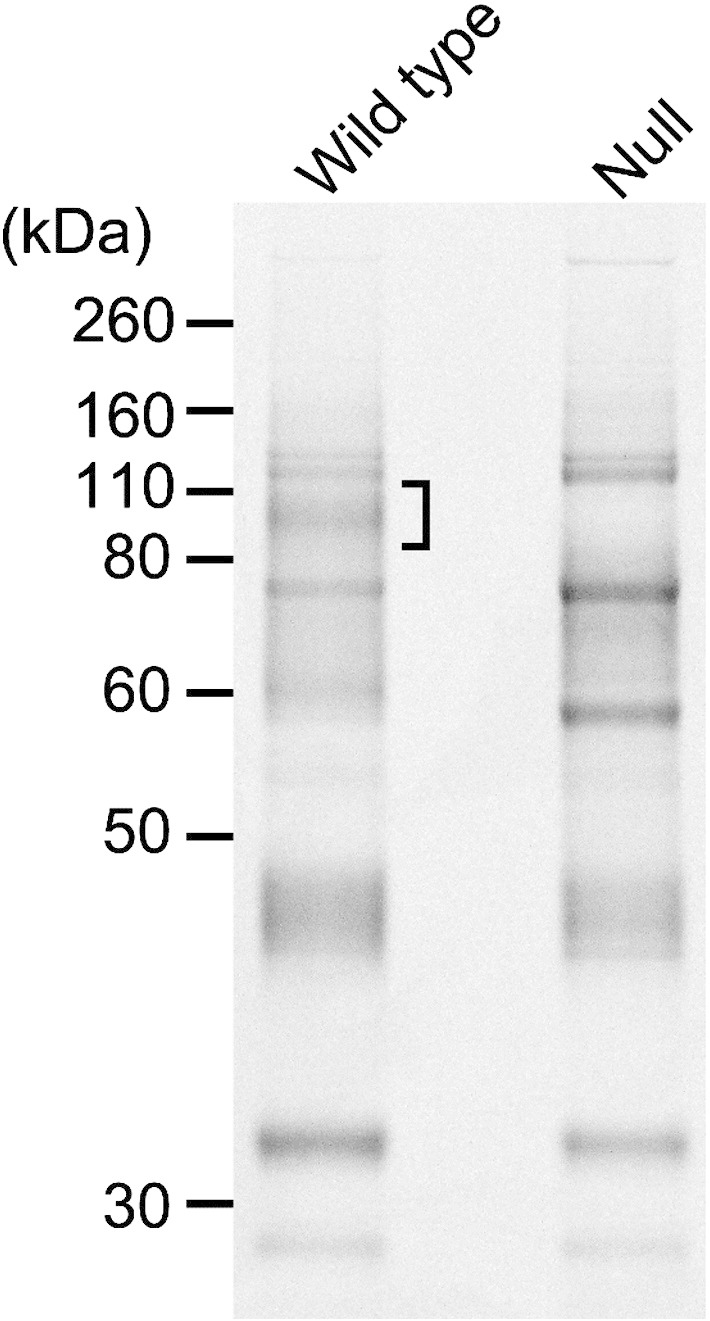
A lectin pull-down showed the loss of the 110-kDa smear band in *TbGT8* null lysates. The parasite lysates were subjected to tomato lectin pull-down experiments and were subsequently eluted competitively with chitin hydrolysate. Wild type and *TbGT8* null strain eluates were separated by SDS–PAGE and stained with Coomassie brilliant blue (CBB). The single bracket indicates the position of the lost glycoprotein smear in the *TbGT8* null lysate.

To identify which glycoproteins bound to tomato lectin in a TbGT8-dependent fashion, tomato lectin-binding glycoproteins were affinity purified from RIPA (−) extracts as described in the Materials and methods. By comparing the Coomassie staining profiles of the chitin hydrolysate eluates of tomato lectin-binding glycoproteins from the wild type and null mutant cell extracts, we observed the loss of a broad protein band with an approximate molecular weight of 110 kDa in the null mutant (Fig. 2). This difference corresponded well with the pattern in Fig. 1. This protein band was excised from the wild-type lane and subjected to proteomic analysis by LC–MS/MS. A MASCOT database search returned a list of proteins, and the top 5 proteins according to the Molecular Weight Search (MOWSE) scores are shown in Table 1. All of the proteins contain 7–14 putative *N*-glycosylation sequons (Asn-Xaa-Ser/Thr, where Xaa cannot be Pro) and have theoretical molecular weights below 110 kDa. In addition, proteins except ESAG2 and HSP83 possess signal sequences at their N-termini, suggesting that they are membrane and/or secretory proteins. ESAG2 is predicted to be a GPI-anchored protein by PredGPI [24], despite the lack of an obvious N-terminal peptide. Thus, we considered that 4 of the 5 candidates were likely to be modified by *N*-linked glycans, allowing the potential for tomato lectin binding. In fact, AcP115 and p67 lysosomal/endosomal proteins have already been reported to be heavily glycosylated proteins with C-terminal transmembrane domains [25,26], and ESAG2 was previously described as able to bind to tomato lectin [16]. Furthermore, a serine carboxypeptidase III homologue was previously reported to be a lysosomal protein with *N*-linked glycosylation in *T. cruzi* [27]. We focused on AcP115 for the following studies because large *N*-linked glycan modifications were expected, given the marked difference between the theoretical (43.7 Da) and apparent molecular weights.

### 3.3 Glycosylation of AcP115 by TbGT8

**Fig. 3:**
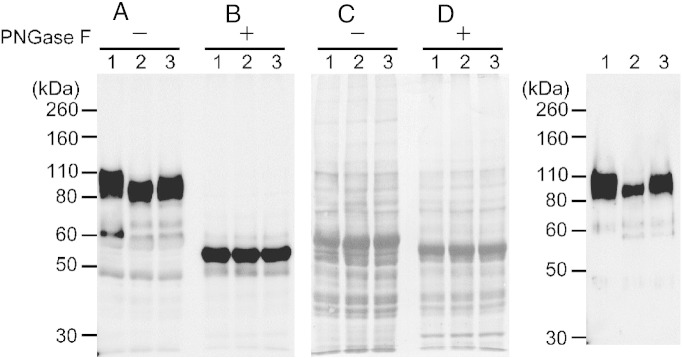
*TbGT8* deletion reduces the size and lectin affinity of AcP115. Panel A: The PVDF membrane used in Fig. 1 was reprobed with an anti-AcP115 antibody. Panel B: CBB staining of the same membrane as the loading control. Panel C: Glycoproteins were pulled down from the lysates with tomato lectin-conjugated agarose and detected with an anti-AcP115 antibody. Lanes 1–3 are the samples from wild type, cKO under non-permissive, and cKO under permissive conditions, respectively. *TbGT8* elimination caused a shift in the molecular weight of AcP115 from approximately 110 kDa to 100 kDa. Only a small amount of AcP115 was included in the pull-down fraction from the parasite under non-permissive conditions.

To investigate whether the loss of TbGT8 expression affected AcP115 glycosylation, the parasite lysates were examined by Western blotting. The anti-AcP115 antibody detected a broad band at approximately 110 kDa in the wild type lysate and a 55-kDa band in the PNGase F-de-*N*-glycosylated lysate (Fig. 3A). These results were fully consistent with those of a previous report by Bakalara et al. [25]. In contrast, AcP115 in the cKO lysate obtained under non-permissive conditions was observed at approximately 100 kDa (Fig. 3A, lane 2). This alteration was restored when the parasite was cultivated under permissive conditions (Fig. 3A, lane 3). Therefore, the lack of TbGT8 accounted for the small shift (approximately 10 kDa) in the molecular size. Furthermore, all AcP115 species converged to 55 kDa and lost reactivity to tomato lectin (Fig. 1) in response to the de-*N-*glycosylation. Therefore, the approximate 10-kDa reduction in the size of AcP115 was accounted for by shortening of *N*-linked glycans. Such a modest alteration in size implies that TbGT8 acts on glycans during a rather late stage of glycan synthesis, as the premature termination of glycan synthesis at an early stage should result in larger shifts in the molecular size. Similarly, the lack of TbGT8 should affect the terminal structures of glycans and thus yield a reduced affinity of AcP115 for tomato lectin. To confirm this, the lectin-binding molecules were pulled down from the lysates with tomato lectin-conjugated agarose and detected with an anti-AcP115 antibody. Despite the comparable amounts of AcP115 in the lysates, the amount of AcP115 in the pull-down fraction was evidently reduced in the non-permissive parasite (Fig. 3C). The AcP115 with the short *N*-linked glycans was not observed as a band in the gel (Fig. 2, lane Null), because the amount was too small to detect with CBB staining. This finding supported the idea that TbGT8-enhanced *N-*glycan(s) on AcP115 contribute to the affinity for tomato lectin.

### 3.4 Glycosylation of TbGT8HA

**Fig. 4:**
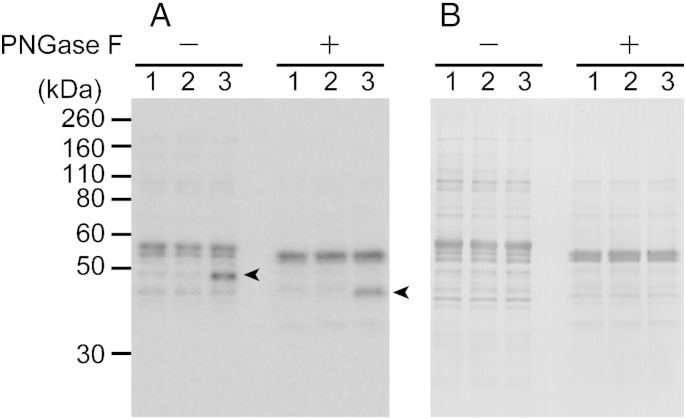
Expression and *N*-linked glycosylation of TbGT8HA. Lanes 1–3 are the samples from wild type, cKO under non-permissive, and cKO under permissive conditions, respectively. Panel A: Immunoblot detection with an anti-HA antibody. Panel B: CBB staining of the same membrane as the loading control. Arrowheads indicate the position of TbGT8HA. De-*N*-glycosylation resulted in a decrease in the molecular weight.

The induced protein expression of TbGT8HA was examined by Western blotting with an anti-HA antibody. TbGT8HA was detected only in the cKO lysates that were cultivated under permissive conditions (Fig. 4). The molecular weight of TbGT8HA shifted from 49 kDa–45 kDa in response to the de-*N-*glycosylation, suggesting that TbGT8HA was also glycosylated. This finding was not surprising because TbGT8 contains a transmembrane region at the N-terminus and 8 potential *N-*glycosylation sites in the subsequent amino acid sequence. This type II transmembrane protein is likely to be glycosylated in the endoplasmic reticulum (ER) and Golgi. This fact led us to investigate the subcellular localization of TbGT8HA.

### 3.5 Colocalization of TbGT8HA with TbGRASP

**Fig. 5:**
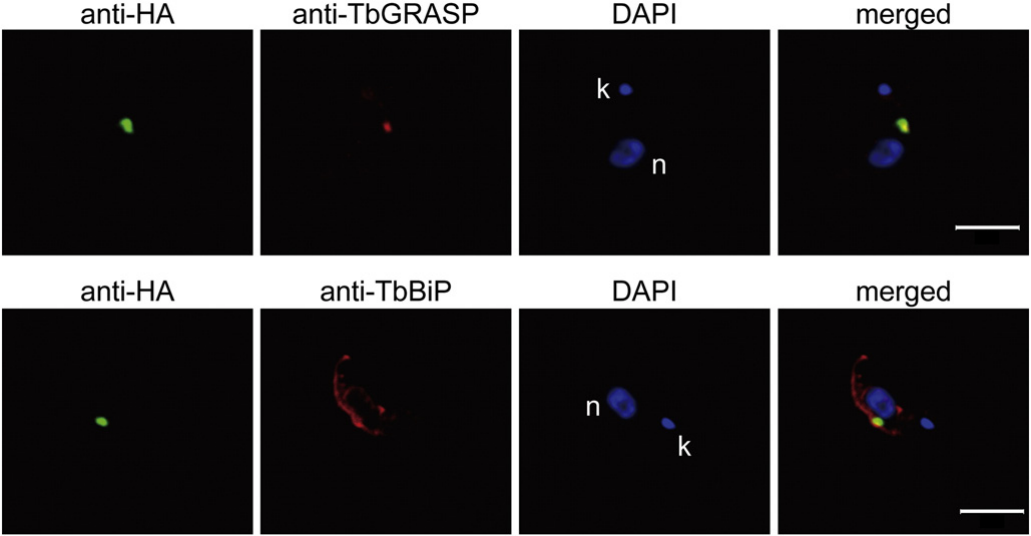
TbGT8HA colocalizes with TbGRASP. The cKO strain under permissive conditions was fixed and double-labeled with anti-HA (green) and either anti-TbGRASP (red) or anti-TbBiP (red). The nucleus (n) and kinetoplast (k) were counterstained with DAPI (blue). Scale bar: 5 μm.

The subcellular localization of TbGT8HA was investigated by indirect immunofluorescent antibody staining of the fixed parasites, using polyclonal antibodies against the respective Golgi and ER marker proteins TbGRASP and TbBiP (Fig. 5). According to confocal fluorescence microscopy, the anti-TbGRASP antibody labeled a specific site near the nucleus, and the anti-TbBiP antibody primarily stained around the nuclei; these were typical patterns of the Golgi and ER, respectively. The HA-tagged protein was detected between the nucleus and kinetoplast and was overlapped with that of TbGRASP but not with TbBiP. Taken together, these data demonstrated that TbGT8 was a Golgi resident enzyme.

## 4 Discussion

TbGT8, a β-1,3-GlcNAc transferase, is involved in the synthesis of branched poly-LacNAc chains that modify the procyclin GPI-anchor in the procyclic form of *T. brucei* [22]. However, the function of TbGT8 in the bloodstream form was unknown, because of the lack of such GPI-anchor modification in this form [6,7,9,14]. In this paper, we revealed a role for TbGT8 in the enhancement of AcP115 *N*-linked glycans in the bloodstream form. The first line of evidence was the decreased molecular weight of AcP115 in the cKO strain under non-permissive conditions. The molecular weight shift is thought to result from immature glycan synthesis due to the lack of TbGT8. The glycan structures are governed by the order and activity of the glycosylhydrolases and glycosyltransferases that process the nascent *N-*linked glycans. The strict specificities of glycosyltransferases for the donor and acceptor substrates often prohibit other transferases from compensating for the lack of a glycosyltransferase [2], resulting in immature and truncated glycans. Because TbGT8 transfers GlcNAc units to the non-reducing end of Gal, the truncated glycan is expected to contain fewer LacNAc repeats. This reduction in mass correlates with a significant decrease in the amount of AcP115 that can bind to tomato lectin–agarose, which primarily recognizes LacNAc glycans (Fig. 3C). As indicated in [28], tomato lectin can also bind to oligomannose *N-*glycans [29] and it is therefore possible that AcP115 may contain TbGT8-independent but tomato lectin-recognizable structures, as well as TbGT8-dependent structures.

In contrast, the tomato lectin blot revealed a broad band at approximately 110 kDa, compared with the Western blot with anti-AcP115, suggesting the existence of other glycoproteins that can bind to tomato lectin and are modified by TbGT8. During the course of this study, 4 other candidates were identified from the comparative proteomic analysis: p67, heat shock protein 83, serine carboxypeptidase III, and ESAG2. These proteins are likely to be modified by *N*-linked glycans allowing the potential to become positive for tomato lectin binding. p67 lysosomal protein has been extensively studied with respect to lysosome trafficking and is suggested to contain poly LacNAc glycan chains. Our overall conclusion is that TbGT8 governs the synthesis of the high-affinity glycan epitopes (most likely poly-LacNAc structures) that are responsible for some of the tomato lectin binding observed in the bloodstream form of *T. brucei*.

In a previous paper, we reported that TbGT8 was non-essential for in vitro growth and infections in mice [22]. TbGT8-dependent glycosylations of target glycoproteins have limited effects on the parasite's growth and survival. The physiological role of AcP115, a target of TbGT8-dependent glycan processing, is currently unknown, but a recombinant protein expressed in *E. coli* exhibited phosphatase activity against phosphorylated peptides and ATP in vitro [25]. Because *E. coli* do not add *N*-linked glycans to their proteins, *N*-linked glycans on AcP115 would appear to have a limited or no impact on enzymatic activity. The non-essential phenotype of TbGT8 with respect to growth may reflect this fact.

Linear poly LacNAc-containing *N*-glycans have been suggested to be an internalization signal for endocytosis in trypanosomes [16]. In this model, a linear poly LacNAc chain is bound to an FP protein that possesses a tomato lectin-like binding domain and a cytoplasmic domain that interacts with the internal endocytic pathway machinery. However, questions about this model were raised by a recent study [14] on the transferrin receptor, a FP-residing glycoprotein in the bloodstream form of *T. brucei*. The study did not find any poly-LacNAc on the polypeptide, despite the ability of poly-LacNAc to undergo endocytic internalization [14]. The significant reductions in tomato lectin-binding glycans without apparent effects on cell growth in the bloodstream form of the *TbGT8* mutant, described herein, and in a single-subunit oligosaccharyltransferase (TbSTT3A)-knockdown cell line, described in [30], also lead to questions regarding the essential role for poly-LacNAc structures in endocytosis and receptor-mediated nutrient uptake.

The conditional expression of HA-tagged TbGT8 revealed its subcellular localization to the Golgi and modification by *N*-linked glycan(s). In general, the synthesis of complex-type *N*-linked glycans such as poly LacNAc occurs in the Golgi, and therefore it is reasonable to detect glycosylated TbGT8 in the Golgi. Simultaneously, this finding suggests that the Golgi is the modifying platform for GPI-linked glycans, as well as for the complex type *N-*linked glycans. The GPI-anchors of *T. brucei* are unusually modified by glycan side chains attached to the trimannosyl core residues. Branched poly LacNAc/lacto-*N-*biose and oligogalactose structures decorate the GPI cores of the procyclic and bloodstream forms, respectively. TbGT8 is also involved in the formation of branched poly LacNAc/lacto-*N*-biose. One reason why this variation exists, despite TbGT8 expression in both lifecycle forms, may be the differential expression of other UDP-Gal/GlcNAc dependent transferases that create TbGT8 acceptor substrates in either a GPI or *N-*glycan context.

In conclusion, our findings revealed that TbGT8 resides in the Golgi and acts as a bifunctional glycosyltransferase that is involved in the synthesis of GPI-linked and *N*-linked glycans in the procyclic and bloodstream forms of *T. brucei*, respectively. With respect to the bloodstream form *N-*glycans, the stage-specific glycoprotein AcP115 is a target of TbGT8-dependent glycosylation.

## Figures and Tables

**Table 1:** List of identified proteins from the smear band near 100 kDa.

Gene ID	Protein name	Protein mass	Number of sequons
		(kDa)	
Tb927.5.630	Acidic phosphatase	43.7	8
Tb927.5.1810	p67 lysosomal/endosomal protein	72.7	14
Tb11.01.6230	Expression site associated-gene 2	55.5	7
Tb927.10.10980	Heat shock protein 83	80.8	7
Tb927.10.1040	Serine carboxypeptidase III precursor	51.5	7
